# Cardiovascular Risk Factors in Women With Primary Sjögren's Syndrome: United Kingdom Primary Sjögren's Syndrome Registry Results

**DOI:** 10.1002/acr.22227

**Published:** 2014-04-22

**Authors:** M Juarez, T E Toms, P de Pablo, S Mitchell, S Bowman, P Nightingale, E J Price, B Griffiths, J Hunter, M Gupta, M Bombardieri, N Sutcliffe, C Pitzalis, C Pease, J Andrews, P Emery, M Regan, I Giles, D Isenberg, R Moots, K S Collins, W F Ng, G D Kitas, on behalf of the UK PRIMARY SJÖGREN'S SYNDROME REGISTRY

**Affiliations:** 1 University of BirminghamBirmingham, UK; 2 Dudley Group of Hospitals NHS TrustDudley, UK; 3 Newcastle UniversityNewcastle upon Tyne, UK; 4 University Hospital BirminghamBirmingham, UK; 5 Great Western Hospitals NHS Foundation TrustSwindon, UK; 6 Gartnavel General HospitalGlasgow, UK; 7 Barts and the London NHS TrustLondon, UK; 8 University of Leeds & NIHR Leeds Musculoskeletal Biomedical Research Unit, Leeds Teaching Hospitals TrustLeeds, UK; 9 Derbyshire Royal Infirmary NHS Foundation TrustDerby, UK; 10 University College London Hospitals NHS Foundation TrustLondon, UK; 11 Aintree University Hospitals NHS Foundation TrustLiverpool, UK; 12 Dudley Group of Hospitals NHS Trust, Dudley, UK, and Manchester UniversityManchester, UK

## Abstract

**Objective:**

To determine the prevalence of traditional cardiovascular risk factors using established definitions in a large cohort of clinically well-characterized primary Sjögren's syndrome (SS) patients and to compare them to healthy controls.

**Methods:**

Data on cardiovascular risk factors in primary SS patients and controls were collected prospectively using a standardized pro forma. Cardiovascular risk factors were defined according to established definitions. The prevalence of cardiovascular risk factors in the primary SS group was determined and compared to that in the control group.

**Results:**

Primary SS patients had a higher prevalence of hypertension (28–50% versus 15.5–25.6%; *P* < 0.01) and hypertriglyceridemia (21% versus 9.5%; *P* = 0.002) than age- and sex-matched healthy controls. Furthermore, a significant percentage (56%) of hypertensive patients expected to be on antihypertensive treatment according to best practice was not receiving it.

**Conclusion:**

Primary SS patients are more than 2 times more likely to experience hypertension and hypertriglyceridemia than age- and sex-matched healthy controls. Additionally, hypertension is underdiagnosed and suboptimally treated in primary SS.

## INTRODUCTION

The association between cardiovascular risk and chronic multisystem inflammatory conditions has been the subject of a large body of research. There is compelling evidence of increased cardiovascular mortality and morbidity in conditions such as rheumatoid arthritis (RA) (1–3) and systemic lupus erythematosus (SLE) (4). In RA, the excess cardiovascular risk is attributed to a combination of traditional and novel cardiovascular risk factors (5) and is a key contributor to the overall mortality and morbidity of this condition. Patients with RA have an increased cardiovascular risk that is comparable to that of patients with diabetes mellitus (6). These observations have led to the recommendation of annual cardiovascular risk assessment for all RA patients (7).

Primary Sjögren's syndrome (SS) is an autoimmune disorder characterized by chronic multisystem inflammation with shared pathophysiology with SLE and RA. However, the links between primary SS and cardiovascular disease (CVD) remain unclear. Five studies have reported on the prevalence of traditional cardiovascular risk factors in primary SS patients (8–11), with 3 of them primarily designed for this purpose (8,9,12). The results of these studies differ. Lodde et al (12) investigated the lipid profile of 46 primary SS patients and found lower high-density lipoprotein (HDL) and total cholesterol levels than in xerostomic controls (n = 12). In contrast, Cruz et al reported similar lipid profiles in primary SS patients (n = 73) and controls (n = 65) (8). More recently, Perez de Lis et al reported that diabetes mellitus and hypertriglyceridemia are more prevalent in primary SS than in primary care patients in Spain (9). These studies have used different methodologies and definitions for cardiovascular risk factors and have reached varying conclusions.

In this study, we determined the prevalence of traditional cardiovascular risk factors using established definitions in a large well-characterized primary SS patient cohort in the UK (13).

**Significance & Innovations**

- Cardiovascular risk is not routinely assessed in primary Sjögren's syndrome (SS) patients, while it is in other chronic autoimmune conditions such as rheumatoid arthritis.
- We set out to determine whether primary SS patients had a higher prevalence of cardiovascular risk factors than healthy controls.
- Female primary SS patients are more than 2 times more likely to experience hypertension and hypertriglyceridemia than age-matched healthy controls.
- Hypertension in primary SS is underdiagnosed and undertreated.

## PATIENTS AND METHODS

### Study subjects

Data from 543 patients with primary SS (5 males, 538 females) recruited to the UK Primary Sjögren's Syndrome Registry (UKPSSR) cohort (13) from 30 centers were analyzed. All patients fulfilled the American-European Consensus Group (AECG) criteria for the classification of primary SS (14). Data on cardiovascular risk were prospectively collected as a predetermined substudy of the UKPSSR (13).

Data for the healthy control group (n = 473; 63 males, 410 females) were derived from 2 cohorts. A parallel control group was recruited alongside the UKPSSR cohort using a “find a friend approach” (n = 111). Briefly, patients were asked to bring a friend (non–blood relative) to their research clinic visit and data were collected from these healthy individuals as well as from the patients. These controls were age and sex matched to the primary SS patients. The second control group (n = 362) came from a study undertaken at Great Western Hospital NHS Foundation Trust, UK with the primary aim to analyze the relationship between C-reactive protein (CRP) and obesity in healthy individuals.

All studies had Research Ethics Committee approval and participants gave written consent according to the Declaration of Helsinki. Data collection was standardized for patients and controls. As only 5 of the primary SS patients were male, subsequent analysis was performed on female participants only.

### Definitions of traditional cardiovascular risk factors

Traditional cardiovascular risk factors were defined according to established criteria. Hypertension was classified using the following World Health Organization (WHO) definitions (15): systolic blood pressure (SBP) ≥140 mm Hg or diastolic blood pressure (DBP) ≥90 mm Hg, and the following National Cholesterol Education Programme (NCEP) definitions (16): SBP ≥130 mm Hg or DBP ≥85 mm Hg or receiving antihypertensive treatment. Abnormal lipid values were defined according to the following NCEP guidelines (16): total cholesterol ≥6.2 mmoles/liter, low-density lipoprotein (LDL) ≥4.13 mmoles/liter, HDL <1.03 mmoles/liter, and triglycerides ≥1.7 mmoles/liter. Obesity was defined as a body mass index (BMI) ≥30 kg/m^2^ (15). Smoking was categorized as current, ex-smoker, and never smoked. Family history of CVD and the presence of diabetes mellitus were assessed from medical records and clinical consultation. Blood pressure was the mean of 3 measurements taken at 5-minute intervals after at least a 5-minute rest. Fasting (≥8 hours) blood glucose, lipid profile (total cholesterol, triglycerides, HDL, and LDL), and CRP levels were measured using routine laboratory methods.

### Cardiovascular events

Self-reported cardiovascular events were recorded at recruitment. Events included myocardial infarction (MI), transient ischemic attacks and/or strokes (collectively referred to as strokes), and peripheral vascular disease.

### Autoantibody test

Anti-Ro and anti-La antibodies were measured in a single laboratory (Newcastle Hospitals NHS Foundation Trust) using the Euroassay anti-ENA Profile Plus according to manufacturer's protocol (Euroimmun AG).

### Statistical analysis

All analyses were carried out with PASW 18.0 (SPSS). Values were expressed as percentages, means, and medians as appropriate. Comparisons were done using chi-square test and Fisher's exact test for categorical data and Mann-Whitney test for nonparametric continuous data. Logistic regression was used to adjust for confounders. Subset analysis was carried out in age-matched primary SS patients and healthy controls. Patients and healthy controls were matched 1:1 on age, rounded to the nearest year. A random number was generated for each individual using Microsoft Excel, and for each age those individuals in the available pool of possible exact matches with the lowest random numbers were selected until sufficient matches were obtained.

## RESULTS

**Figure 1:**
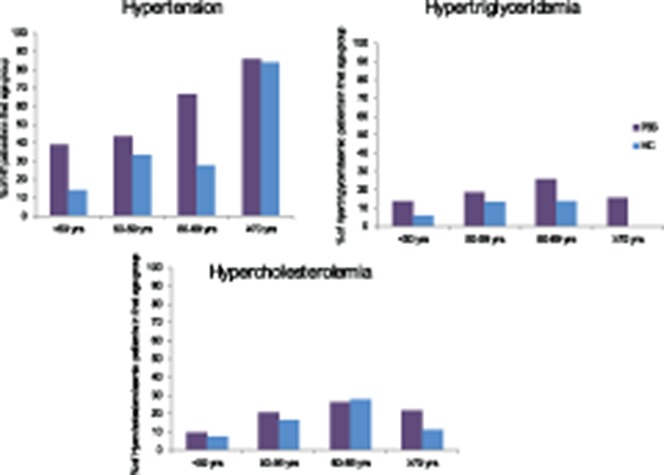
Distribution of hypertension (HT), hypertriglyceridemia, and hypercholesterolemia in primary Sjögren's syndrome (PSS) patients and healthy controls (HC) by age group.

Data from 538 female primary SS patients fulfilling the AECG criteria for the classification of primary SS were included. Patients' mean ± SD age was 59 ± 12.4 years. All patients and controls were white. Table 1 summarizes the demographic characteristics and prevalence of traditional cardiovascular risk factors in patients and controls. The prevalence of hypertension, hypercholesterolemia, and hypertriglyceridemia was higher among primary SS patients compared to healthy controls. There was also higher frequency of primary SS patients taking antihypertensive and statin therapies. Primary SS patients had lower proportions of current smokers and oral contraceptive users than healthy controls. Thirty percent of patients were taking hydroxychloroquine, 10% were taking steroids, and 6% were taking immunosuppressants, with none of the controls taking any of these medications. Patients were younger than controls (mean ± SD age 45.8 ± 11.8 versus 59.1 ± 12.4 years; *P* < 0.001). Since age is a key determinant of blood pressure and serum lipid levels, we stratified the prevalence of hypertension, hypertriglycemia, and hypercholesterolemia according to age and demonstrated that the percentages of primary SS patients with hypertension or hypertriglyceridemia were higher than healthy controls in all age categories. The same was true for hypercholesterolemia, except for the 60–69 years age group, where the percentage of patients with hypercholesterolemia was higher in the control group (Figure 1).

**Table 1:** Demographics and prevalence of cardiovascular risk factors in primary SS patients and healthy controls*

	Primary SS patients (n = 538)	Healthy controls (n = 410)
Age, mean ± SD years	59.1 ± 12.4	45.8 ± 11.8
Smoking, current	22 (4)	33 (8)
WHO hypertension	203 (37.7)	43 (10.4)
NCEP hypertension	308 (57.3)	94 (23)
Hypercholesterolemia	95 (17.7)	60 (14.5)
Hypertriglyceridemia	127 (23.6)	36 (8.7)
Low HDL	70 (13.1)	43 (12.1)
High LDL	71 (13.2)	55 (15.6)
Diabetes mellitus	19 (3.5)	7 (1.7)
Family history of CVD	67 (12.5)	51 (12.7)
CRP, median (IQR) mg/dl	5.0 (2.7–7.9)	1.15 (0.0–3.0)
BMI ≥30 kg/m^2^	110 (20.4)	67 (16.2)
Antihypertensive use	150 (27.8)	41 (10)
Statin use	84 (15.6)	15 (3.6)
Fibrate use	1 (0.2)	0 (0)

* Values are the number (percentage) unless indicated otherwise.

SS = Sjögren's syndrome; WHO = World Health Organization; NCEP = National Cholesterol Education Programme; HDL = high-density lipoprotein; LDL = low-density lipoprotein; CVD = cardiovascular disease; CRP = C-reactive protein; IQR = interquartile range; BMI = body mass index.

### Subset analysis

In order to further investigate whether the increased prevalence of hypertension and dyslipidemia was a consequence of the younger age of the control group, we examined a group of 200 primary SS patients and 200 controls that could be matched for age, sex, and ethnicity. Supplementary Table 1 (available in the online version of this article at http://onlinelibrary.wiley.com/doi/10.1002/acr.22227/abstract) compares the characteristics of primary SS patients included in this subset analysis with the remaining primary SS cohort. As expected, patients included in the subset analysis were younger. In addition, this subset of primary SS patients also had a lower prevalence of hypertension, with smaller proportions of individuals receiving antihypertensive or statin therapies, but with higher percentages of subjects taking antimalarials and oral contraceptives.

The mean ± SD age of patients and controls in the subset analysis was 51 ± 10 years. A total of 88.4% of patients were anti-Ro antibody positive and 68.9% were anti-La antibody positive with a mean ± SD disease duration of 11.5 ± 8.5 years from symptom onset.

Primary SS patients had a lower prevalence of smoking than healthy controls (3.8% versus 10.1%; *P* = 0.026) and higher median levels of CRP (5 mg/dl versus 1.2 mg/dl; *P* < 0.0001). There was no difference in the prevalence of diabetes mellitus, obesity, or family history of CVD. There was no correlation between steroid use and diabetes mellitus prevalence in either group. Analysis of lipid profiles revealed that 21% of patients had hypertriglyceridemia compared to 9.5% of controls (*P* = 0.002), but there was no statistically significant difference in the prevalence of hypercholesterolemia, low HDL, or high LDL levels (Table 2). Hypertension was more prevalent in primary SS patients than in controls regardless of the definition used. Twenty-eight percent of patients were classified as having WHO hypertension compared to 15% of controls (*P* = 0.003), while 50% of patients were classified as having NCEP hypertension versus 25.6% of controls (*P* < 0.0001).

**Table 2:** Prevalence of traditional cardiovascular risk factors in age-matched primary SS patients and healthy controls*

	Primary SS patients (n = 200)	Healthy controls (n = 200)	*P*
Smoking	7 (3.8)	20 (10.1)	0.026
WHO hypertension	56 (28)	31 (15.5)	0.003
NCEP hypertension	100 (50)	51 (25.6)	< 0.0001
Hypercholesterolemia	38 (19)	35 (17.5)	0.796
Hypertriglyceridemia	42 (21)	19 (9.5)	0.002
Low HDL	33 (16.5)	26 (13)	0.398
High LDL	33 (16.5)	37 (18.5)	0.693
Diabetes mellitus	6 (3)	4 (2)	0.543
Family history of CVD	31 (15.5)	34 (17)	0.398
CRP, median (IQR) mg/dl	5.0 (2.7–7.0)	1.2 (0.0–2.8)	< 0.0001
BMI ≥30 kg/m^2^	39 (19.5)	37 (18.6)	0.899
Antihypertensive use	40 (20)	28 (14)	0.143
Statin use	12 (6)	9 (4.5)	0.655
Fibrate use	0 (0)	0 (0)	1.000

* Values are the number (percentage) unless indicated otherwise.

SS = Sjögren's syndrome; WHO = World Health Organization; NCEP = National Cholesterol Education Programme; HDL = high-density lipoprotein; LDL = low-density lipoprotein; CVD = cardiovascular disease; CRP = C-reactive protein; IQR = interquartile range; BMI = body mass index.

Odds ratios were calculated to determine the likelihood of experiencing hypertriglyceridemia and hypertension after adjusting for smoking, BMI, and steroid, antimalarial, and antihypertensive use where appropriate. Primary SS patients were 2.260 (95% confidence interval [95% CI] 1.262–4.080, *P* = 0.006) times more likely to have hypertriglyceridemia, 2.02 (95% CI 1.19–3.44, *P* = 0.009) times more likely to have WHO hypertension, and 2.87 (95% CI 1.83–4.52, *P* < 0.0001) times more likely to have NCEP hypertension than controls.

### Characteristics of patients with hypertension and hypertriglyceridemia

We then analyzed the clinical, laboratory, and immunologic characteristics of hypertensive and nonhypertensive patients to explore whether hypertensive patients could be identified on the basis of any of these characteristics. The only statistically significant difference found was antihypertensive use, with 28% of hypertensive patients on treatment compared to 12% of nonhypertensive patients (*P* = 0.007). The same analysis was carried out for hypertriglyceridemia and nonhypertriglyceridemia primary SS patients and is shown in Table 3. Hypertriglyceridemia primary SS patients were more likely to have abnormal salivary flow test findings (95.2% versus 81.3%; *P* = 0.003), to have positive antinuclear antibodies (ANAs; 90.6% versus 70.6%; *P* = 0.02), and to be taking antihypertensives (*P* < 0.0001) than their nonhypertriglyceridemia counterparts. Of note, neither steroid nor hydroxychloroquine use influenced the prevalence of hypertension or hypertriglyceridemia.

**Table 3:** Clinical and laboratory characteristics of hypertriglyceridemia vs. non-hypertriglyceridemia primary SS patients*

	Hypertriglyceridemia (n = 42)	Non- hypertriglyceridemia (n = 158)	*P*
Abnormal Schirmer's test	22 (52.4)	83/157 (52.9)	1.000
Abnormal salivary flow test	40 (95.2)	126/155 (81.3)	0.030
Abnormal parotid sialography	4/5 (80.0)	9/18 (50.0)	0.339
Positive salivary gland biopsy	11/17 (64.7)	39/53 (73.6)	0.543
CRP, median (IQR)	3.25 (1.7–5.5)	1.5 (0–4.5)	0.477
Positive anti-Ro	35/41 (85.3)	140/157 (89.1)	0.583
Positive anti-La	28/41 (68.2)	105/157 (66.8)	0.704
Positive ANA	29/32 (90.6)	77/109 (70.6)	0.021
Positive RF	23/32 (71.9)	76/110 (69.1)	0.830
Antihypertensive use	18 (42.9)	22 (13.9)	< 0.0001
Statin use	2 (4.7)	10 (6.3)	1.000
Steroid use	6 (14.3)	9 (5.6)	0.092
Antimalarial use	14 (33.3)	59 (37.3)	0.720
Fibrate use	0 (0)	0 (0)	1.000

* Values are the number (percentage) or the number/total number (percentage).

SS = Sjögren's syndrome; CRP = C-reactive protein; IQR = interquartile range; ANA = antinuclear antibody; RF = rheumatoid factor.

### Management of hypertension in primary SS patients

Forty of the 200 primary SS patients from the subset analysis were known to have hypertension and were taking antihypertensive treatment. Out of these 40 patients on treatment, 12 had controlled hypertension, while the remaining 28 remained hypertensive despite medication (inadequately controlled hypertension). These findings are summarized in Table 4.

**Table 4:** Blood pressure values and antihypertensive treatment in primary SS patients and controls included in the subset analysis*

	Normotension (<135/85 mm Hg)	Hypertension (≥135/85 mm Hg)	Stage 2 hypertension (≥160/100 mm Hg)
Primary SS patients, no.			
Taking antihypertensives	12	28	7
Not taking antihypertensives	88	72	9
Total	100	100	16
Healthy controls, no.			
Taking antihypertensives	10	18	3
Not taking antihypertensives	139	33	1
Total	149	51	4

* SS = Sjögren's syndrome.

Therefore, only 28 of the 100 hypertensive primary SS patients identified had prior knowledge of their condition, with 72 primary SS patients being diagnosed as hypertensive during the course of this study. In order to determine how many of these patients should be taking antihypertensive treatment, we applied the National Institute for Health and Care Excellence guidance on clinical management of hypertension in adults (17). For patients with hypertension, the need for drug treatment (in addition to lifestyle modifications) depends on the absolute blood pressure value, patient's age, and comorbidities. However, if SBP ≥160 mm Hg or DBP ≥100 mm Hg (stage 2 hypertension), drug treatment is warranted regardless of comorbidities if ambulatory blood pressure monitoring confirms hypertension (16). Applying this guidance to our cohort, 16 of the 100 hypertensive primary SS patients would be expected to be on drug treatment. To our surprise, only 44% of these patients were taking antihypertensive agents (and were therefore experiencing inadequately controlled hypertension), while the other 56% were not receiving antihypertensive drugs (and were therefore experiencing untreated hypertension). When the same cutoff was applied to the healthy controls, we identified 4 patients requiring treatment. Of these, 3 patients were taking medication and 1 was not.

### Prevalence of cardiovascular events

A total of 7 cardiovascular events were recorded in the primary SS patients included in the subset analysis. One patient had an MI, while another 6 patients experienced strokes. There were no recorded peripheral vascular disease events. All 7 patients had positive anti-Ro and anti-La antibody test findings and experienced hypertension (4 controlled, 3 uncontrolled). None of the patients were smokers, 2 of them experienced hypertriglyceridemia, and 1 experienced hypercholesterolemia. Information on cardiovascular events was not available for healthy controls and therefore direct comparisons between groups could not be drawn.

## DISCUSSION

We have determined the prevalence of traditional cardiovascular risk factors, using established definitions, in a large multicenter cohort of white female primary SS patients in the UK. We found that primary SS patients were more than 2 times more likely to experience hypertension and hypertriglyceridemia than age- and sex-matched healthy controls. Furthermore, a significant proportion of the hypertensive patients were not previously known to experience hypertension, and many of those who should have been on antihypertensive medications were not receiving them. Hypertension in primary SS is therefore underdiagnosed and suboptimally treated.

Hypertension is the most important modifiable risk factor for CVD, being more common than cigarette smoking, dyslipidemia, or diabetes mellitus (18). Hypertension in primary SS may, at least in part, be explained by the increased prevalence of subclinical atherosclerosis (11) seen in these patients. Accelerated atherosclerosis leads to increased cardiovascular events and deaths (19,20) and has been well documented in other chronic inflammatory conditions such as RA (20) and SLE (21). In primary SS, immune-mediated arterial wall damage has been postulated as the underlying cause for accelerated atherosclerosis (11). In RA, age, BMI, and steroid use have been independently associated with hypertension (22). In our study, there was no difference in BMI between primary SS patients and controls, and the effects of age and steroid use were corrected for statistically. Despite this, hypertension was still more prevalent in primary SS patients than in controls. This suggests that the effect of other factors (such as genetic, immunologic, and/or inflammatory) may be more important. Indeed, hypertension has been shown to be associated with specific genetic polymorphisms in RA patients (23,24) and such associations and other potential mechanisms need to be investigated further in primary SS. In apparent contrast with our results, Perez de Lis et al (9) reported a lower prevalence of hypertension in primary SS patients compared to primary care controls. However, it is worth noting that 46% of the individuals in their control group were hypertensive. This led them to hypothesize that their finding may be a paradoxical effect due to a higher prevalence of hypertension in the control group and recognized it as a limitation of their study. Of note, they found 30% of hypertensive patients in their primary SS group, a similar figure to that found in our cohort. In contrast to this study, a significant proportion of the individuals in our control group experienced undiagnosed and sometimes untreated hypertension. While the optimal management of hypertension was not a primary aim of our study and while this does not alter our main finding of a high prevalence of hypertension in primary SS patients, these findings suggest that hypertension may be undertreated both in primary SS and healthy controls.

Data on the lipid profile of primary SS patients are scarce. In the US, Lodde et al (12) reported lower HDL and total cholesterol levels in female primary SS patients (n = 46) than in xerostomic controls (n = 12). They analyzed mean fasting lipid values but did not calculate the prevalence of hypercholesterolemia, hypertriglyceridemia, high LDL, and low HDL using established definitions (16). The raw values of serum lipids of primary SS patients between their study and ours were similar, but different for the control cohorts. Of note, the lipid profile of their xerostomic controls was significantly different from that of the healthy age-matched US population. Our results concur with those of Perez de Lis et al (9), who, using the same definition of hypertriglyceridemia as our study, found a higher prevalence of hypertriglyceridemia in primary SS patients than controls (22% versus 15%; *P* = 0.023). The cause of the abnormal lipid profile in primary SS may be, as in RA (25), multifactorial and, together with its clinical significance, is yet to be fully explored. Corticosteroid use can cause dyslipidemia. However, the prevalence of hypertriglyceridemia was not increased in primary SS patients on steroid treatment compared to those who were not on treatment (data not shown), and the percentage of hypertriglyceridemia primary SS patients taking steroids was not significantly higher than that of their nonhypertriglyceridemia counterparts (14.3% versus 5.6%; *P* = 0.092) (Table 3). Interestingly, we have identified that patients with hypertriglyceridemia are more likely to have abnormal salivary flow test findings and positive ANAs than nonhypertriglyceridemia patients. While these findings need to be validated in different cohorts, this observation may aid stratification of primary SS patients who may benefit from analysis of lipid profile.

Seven primary SS patients experienced cardiovascular events. All of them were hypertensive and had positive anti-Ro and anti-La antibody testing. Information regarding disease duration at the time of cardiovascular events was not collected. The issue of whether cardiovascular risk factors translate into increased cardiovascular risk in primary SS patients is an important one and has not been studied in detail. Perez de Lis et al described cardiovascular events in primary SS patients but reported that their prevalence did not differ from that of the control population (9). We were unable to perform such comparison as we lacked the relevant data for the control group. Nevertheless, in the light of our findings and that of others, a longitudinal study of cardiovascular risk and events in primary SS patients is warranted.

The proportion of current smokers in the primary SS group was significantly lower than that in the control group (3.8 versus 10.1; *P* = 0.02). The reason for this observation is unclear, but has also been reported in another study (9) and may be related to the potential link between smoking and aggravation of xerostomia.

Median CRP levels were higher in primary SS patients than controls. This is in keeping with findings from previous studies (9,12). The association between CVD and inflammation is widely recognized and CRP levels have been used as reliable markers of inflammation. The American Heart Association has defined risk groups for CVD according to high-sensitivity CRP (hsCRP) values (26), where an hsCRP level of <1.0 mg/dl, 1.0–2.9 mg/dl, and ≥3.0 mg/dl confers low, moderate, and high risk, respectively. Although these values are not intended for use in isolation but in conjunction with other cardiovascular risk factors, they act as good indicators of cardiovascular risk. Although CRP levels in our study were measured using routine laboratory tests, the higher values found in the primary SS group support the idea that inflammation may act as an additional and independent risk factor for CVD in primary SS patients.

Perez de Lis et al (9) described a higher prevalence of diabetes mellitus in primary SS patients than in controls. Our study did not confirm this finding. One possible explanation is that the definitions of diabetes mellitus used in these 2 studies were different. However, the magnitude of the difference between studies (3.4% versus 27%) warrants further investigation into the underlying reasons. Glucocorticoid use is a risk factor for diabetes mellitus. In this study, there was no apparent correlation between glucocorticoid use and diabetes mellitus as 2 of 53 (3.8%) primary SS patients taking glucocorticoids and 17 of 485 (3.8%) primary SS patients not on glucocorticoids had diabetes mellitus (*P* = 1.000).

In addition to traditional cardiovascular risk factors, there is now growing interest in the role of nontraditional cardiovascular risk factors. Of particular relevance to primary SS is the potential role of oral inflammation and hygiene as risk factors for cardiovascular events (27,28) since poor oral hygiene and oral mucosal disease are common among primary SS patients. Future studies investigating the link of oral health in primary SS and cardiovascular risk would be of great interest.

The strengths of this study include prospective and standardized data collection, a large sample size, and patient recruitment from multiple centers, which increases the ecological validity of the findings. This study, however, is not without limitations. The control cohort was younger, but our observations of increased prevalence of hypertension and hypertriglycaemia in primary SS patients were confirmed in our subset analysis of age- and sex-matched groups. Some of the individuals in the control cohort were hospital employees. This may have introduced a healthy worker effect bias (29) by which morbidity in this group may be lower than in the general population. However, among the hospital employees, there was a diversity of occupations within this group (nurses [19%], administrative and clerical staff [35%], porters and kitchen workers [17%], doctors [3%], and others [26%]) reflecting a variety of socioeconomic and educational backgrounds. Furthermore, analysis of lifestyle preferences in the control group revealed that more than half the participants did not undertake regular exercise and 60% were ex-smokers. These figures are similar to the general population, arguing against a significant healthy-worker bias in this group.

A large body of research led to the recognition of increased cardiovascular risk in RA. This in turn has led to recommendations of yearly cardiovascular checks in RA patients (7), although adherence to this guideline in daily practice remains suboptimal (30). A similar approach has been proposed in order to assess and treat the increased cardiovascular risk associated with SLE (31). In contrast, cardiovascular risk is not routinely considered during assessment of primary SS patients. Our findings warrant further investigation in this area and will alert clinicians that an association between increased cardiovascular risk and primary SS exists.

Primary SS patients have higher prevalence of hypertension and hypertriglyceridemia than healthy individuals. Clinicians need to be aware of this increased risk in order to take appropriate steps to assess cardiovascular risk and treat it where appropriate.

## AUTHOR CONTRIBUTIONS

All authors were involved in drafting the article or revising it critically for important intellectual content, and all authors approved the final version to be submitted for publication. Dr. Kitas had full access to all of the data in the study and takes responsibility for the integrity of the data and the accuracy of the data analysis.

**Study conception and design.** Bowman, Nightingale, Price, Gupta, Sutcliffe, Andrews, Emery, Isenberg, Ng, Kitas.

**Acquisition of data.** Juarez, Mitchell, Bowman, Nightingale, Price, Griffiths, Hunter, Gupta, Bombardieri, Sutcliffe, Pitzalis, Pease, Andrews, Emery, Regan, Giles, Moots, Ng, Kitas.

**Analysis and interpretation of data.** Juarez, Toms, de Pablo, Bowman, Nightingale, Bombardieri, Sutcliffe, Emery, Moots, Collins, Ng, Kitas.

## APPENDIX A: ADDITIONAL MEMBERS OF THE UK PRIMARY SJÖGREN'S SYNDROME REGISTRY

In addition to Drs. Bowman, Griffiths, and Ng, who are investigators of the UKPSSR, and Drs. Bowman, Price, Ng, and Kitas, who are investigators of the UKPSSR cardiovascular substudy, the other UKPSSR members include, in alphabetical order of their affiliations: Elalaine C. Bacabac, Robert Moots (Aintree University Hospitals); Michele Bombardieri, Constantino Pitzalis, Nurhan Sutcliffe (Bart and the London NHS Trust); Nagui Gendi (Basildon Hospital); John Hamburger, Andrea Richards (Birmingham Dental Hospital); Saaeha Rauz (Birmingham & Midland Eye Centre); Sue Brailsford (Birmingham University Hospital); Paul Allcoat, John McLaren (Whyteman's Brae Hospital, Fife); Joanne Logan Diarmuid Mulherin (Cannock Chase Hospital); Jacqueline Andrews, Paul Emery, Colin Pease, Christine Thomas (Chapel Allerton Hospital, Leeds); Alison Booth, Marian Regan (Derbyshire Royal Infirmary); Esther Gordon, Cathy Lawson (Harrogate District Foundation Trust Hospital); Monica Gupta, John Hunter, Lesley Stirton (Gartnavel General Hospital, Glasgow); Gill Ortiz, Elizabeth Price (Great Western Hospital); Gavin Clunie, Ginny Rose (Ipswich Hospital NHS Trust); Susan Knight, Deborah Symmons, Beverley Jones (Macclesfield District General Hospital & Arthritis Research UK Epidemiology Unit, Manchester); Suzanne Edgar, Marco Carrozzo, Francisco Figuereido, Heather Foggo, Iain Macleod, Dennis Lendrem, Sheryl Mitchell (Newcastle upon Tyne Hospitals NHS Foundation Trust); Adrian Jones, Peter Lanyon, Alice Muir (Nottingham University Hospital); Paula White, Steve Young-Min (Portsmouth Hospitals NHS Trust); Susan Pugmire, Saravanan Vadivelu (Queen's Elizabeth Hospital, Gateshead); Annie Cooper (Royal Hampshire County Hospital); Anne Field, Stephen Kaye, Devesh Mewar (Royal Liverpool University Hospital); Neil McHugh, John Pauling, Julie James (Royal National Hospital for Rheumatic Diseases); Mohammed Akil, Jayne McDermott (Royal Sheffield Hospital); David Coady, Elizabeth Kidd (Royal Sunderland Hospital); Bhaskar Dasgupta, Pamela Long (Southend University Hospital); and Ada Ferenkeh-Koroma, Stefano Fedele, Ian Giles, David Isenberg, Stephen Porter (University College Hospital & Eastman Dental Institute).
