# Supplementary material for: Cardiovascular Risk Factors in Women With Primary Sjögren's Syndrome: United Kingdom Primary Sjögren's Syndrome Registry Results
Source: Arthritis Care Res (Hoboken). 2014 Apr 22;66(5):757–64. doi: 10.1002/acr.22227 (PMC4529667; doi:10.1002/acr.22227)
Supplement: Supplementary file 1 — Supplementary Table 1. [file acr0066-0757-sd1.doc]

**Supplementary Table 1. Comparison of traditional cardiovascular risk factors and concurrent treatment in PSS patients included in subset analysis vs. those not included.**

|  | **PSS patients in subset analysis**  **(n=200)** | **PSS patients not in subset analysis**  **(n=338)** | **p value** |
| --- | --- | --- | --- |
| Age (yrs) | 51.0 (±10) | 63.9 (±11) | <0.001 |
| Smoking current | 7 (3.5) | 15 (4.4) | 0.655 |
| WHO Hypertension | 56 (28) | 147 (43.4) | <0.001 |
| NCEP Hypertension | 100 (50) | 208 (61.5) | 0.009 |
| Hypercholesterolaemia | 38 (19) | 57 (21.5) | 0.562 |
| Hypertriglyceridaemia | 42 (21) | 48 (18.2) | 0.478 |
| Low HDL | 33 (16.5) | 37 (16) | 1.000 |
| High LDL | 33 (16.5) | 38 (16.8) | 1.000 |
| Diabetes | 6 (3) | 13 (3.8) | 0.810 |
| Family history of CVD | 31 (15.5) | 36 (10.6) | 0.106 |
| CRP | 5.0 (2.7-7.0) | 5.0 (2.7-8.2) | 0.477 |
| BMI ≥ 30 | 39 (19.5) | 71 (21) | 0.740 |
| Antihypertensive use | 40 (20) | 110 (32.5) | 0.002 |
| Statin use | 12 (6) | 72 (21.3) | <0.001 |
| Fibrate use | 0 (0) | 1 (0.3) | 1.000 |
| Antimalarials use | 73 (36.5) | 95 (28.1) | 0.044 |
| Steroids use | 15 (7.5) | 38 (11.2) | 0.180 |
| Immunosuppressants use | 12 (6) | 18 (5.3) | 0.846 |
| Rituximab use | 1 (0.5) | 1 (0.3) | 1.000 |
| Contraception use | 6 (3) | 0 (0) | 0.003 |
| HRT use | 14 (7) | 12 (3.6) | 0.095 |

Results expressed as mean (±SD), median (IQR) and values (percentages) as appropriate. PSS: Primary Sjögren’s syndrome, WHO: World Health Organization, NCEP: National Cholesterol Education Programme, HDL: high density lipoprotein, LDL: low density lipoprotein, CVD: cardiovascular disease, BMI: body mass index, HRT: hormone replacement therapy.
